# Evaluation of the relationship between the expression of AgNOR and Ki67 with the recurrence rate in central granulomatous giant cell lesions: A case‐control

**DOI:** 10.1002/cre2.870

**Published:** 2024-03-20

**Authors:** Mina T. B. Dareh, Azadeh Andisheh‐Tadbir, Arezoo Aghakouchakzadeh

**Affiliations:** ^1^ Student Research Committee, School of Dentistry Alborz University of Medical Sciences Karaj Iran; ^2^ Department of Oral and Maxillofacial Pathology, School of Dentistry, Oral and Dental Disease Research Center Shiraz University of Medical Sciences Shiraz Iran; ^3^ Department of oral and maxillofacial pathology, School of Dentistry Alborz University of Medical Science Karaj Iran

**Keywords:** AgNOR, central giant cell granuloma, Ki67, recurrence

## Abstract

**Objectives:**

Giant cell granuloma is a local nonneoplastic lesion that is divided into two categories, based on its site of occurrence: Central and peripheral giant cell granuloma. Central giant cell granuloma is an intraosseous lesion that has a tendency to recure even in surgically treated cases. Several studies have proven that there is an association between different lesions clinical behavior and their histological features. The aim of this study was to evaluate the expression of AgNOR and Ki67 in lesions with and without recurrency.

**Material and Methods:**

Files and records of 35 patients who had been histologically diagnosed with central giant cell granuloma were investigated. Histological features were studied after performing AgNOR staining and Ki67 marker. The data were analyzed by chi‐square, Fisher, and *T*‐test.

**Results:**

Acquired data indicated that the count of AgNOR staining and Ki67 marker was significantly higher in lesions with recurrency than the lesions with no recurrency. The same results were attained from Ki67 intensity.

**Conclusion:**

The current study indicated that AgNOR staining and Ki67 marker have prognostic value in predicting recurrency of central giant cell granuloma lesions.

## 1 INTRODUCTION

Giant cell granuloma (GCG) was first described by Henry L. Jaffe, as a local nonneoplastic reparative reaction of bone that is classified differently from giant cell tumor of bone. Since available lesions at that time were hemorrhagic, it was believed that trauma is the etiology of these lesions. By revealing cases with no trauma history, the etiology of GCGs has remained unknown.

GCG prevalence rate is 1.1 per million per year; but its recurrence rate can be up to 49%, even in surgically treated cases, which make it the primary concern about GCG.

According to the site of the lesion, GCGs are divided into two categories: Central or peripheral. Central GCG (CGCG) is an intraosseous lesion, while peripheral GCG (PGCG) appears in soft tissue (Richardson et al., 2022).

PGCG is a reactive exophytic and extraosseous lesion that occurs in edentulous area of alveolar ridge or in gingiva (Maheshwari et al., 2017). It is a common lesion that can appear in variable sizes and may be pedunculate or sessile with deep red to bluish‐red color and tumor‐like growth (Srivastava et al., 2022).

PGCG is usually a result of local irritation by local factors. Treatment by excision of the lesion with accompaniment of removing local factors, will result in low recurrency rate (Maheshwari et al., 2017).

According to WHO classification, CGCG is a benign nonodontogenic connective tissue neoplasm (Bhat, 2022) that develops in the hard tissue of bone and it has 7% prevalence among jaw tumors (Bocchialini et al., 2020). This lesion usually occurs in the anterior part of mandible and has a tendency among young female patients (Souza et al., 2000) and two‐thirds of cases are patients under 30 years old (Ramesh, 2020). The origin of CGCG is not completely clear. It can be a reactive lesion, a benign neoplasm or a developmental anomaly (Bocchialini et al., 2020). CGCG is asymptomatic in primary phases but, there is a matter of time till it expanses cortical bone (Ramesh, 2020).

The usual treatment of CGCG is surgical excision, which its extension varies from common curettage to en bloc resection. Conservative treatment methods such as intralesional injection of corticosteroids are also available (Kruse‐Lösler et al., 2006).

CGCG and PGCG have similar histological features (Souza et al., 2000). Fibroblasts are main cells in the background and several giant cells are scattered among them. Some features can be seen through cellular connective tissue; including capillaries, hemorrhage, hemosiderin, and inflammatory cells (Srivastava et al., 2022).

CGCGs may differ in their clinical behavior; in some cases, lesion grows slowly and never occurs again. On the other hand, some lesions grow rapidly and they tend to recur (Souza et al., 2000).

Based on clinical and radiographical findings, these lesions can be described in two categories: aggressive and nonaggressive. Aggressive forms have a higher tendency for recurrence (Marti‐Flich et al., 2022). CGCG's global recurrence rate varies in different regions and it has been reported between 11% and 49%. For aggressive‐type lesions, recurrence rate could rise to 70% (De Cidrac et al., 2021).

Several studies have shown that there is an association between lesion's clinical behavior and its histological features (Melo‐Muniz et al., 2020).

Proliferation activity of every tissue or neoplasm can be determined based on its cell proliferation rate. One of the most common indicators for assessment of cell proliferation is Ki67, which is a remarkable marker for determining cell's growth fraction in normal or dysplastic tissue (HM El Attar & Wahba, 2016). This protein was first identified by Scholzer and Gerdes in the early 1980s, as an antigen on Hodgkin lymphoma cells. Ki67 can be detected during all active phases of the cell cycle, which means G_1_, S, G_2_, and M phases. But it is absent during resting phase, G_0_. The prognostic value of Ki‐67 protein has been evaluated in numerous studies, indicating its potential as a reliable marker (Takkem et al., 2018).

The CGCG's fibroblasts are proliferative component of this lesion and they express Ki67 protein which is indicative of the cells in the cell cycle. Also, it is believed that these fibroblasts are responsible for the recruitment and retention of monocytes transforming to multinucleated giant cells (Ramesh, 2020).

Nucleolar organizer regions (NORS) are defined as chromosomal segments containing ribosome coding genes. NORs contain a set of proteins that are able to bind silver ions. The silver‐stained NORs and the NOR‐associated proteins are called AgNORs. In light microscopes, the AgNOR proteins are detected as well‐defined black dots. In interphase, these dots are located within nucleoli. AgNOR protein amount and quantity are valuable parameters for assessment of cell kinetics and they are significantly contributed with cell proliferation rate (Trerè, 2000).

Silver staining of AgNORs is considered as the best and most cost‐effective marker to evaluate the proliferative behavior of a lesion. The cell proliferation rate is evaluated by rapidity of the cell cycle and AgNOR count per nucleus (Chandrashekar et al., 2020).

Recently, the AgNOR method has been widely assessed among pathological experiments, and over 1000 research have been published about diagnostic and prognostic applications of the AgNOR parameters in different fields of tumor pathology (Trerè, 2000).

As we mentioned, recurrence rate of CGCGs is relatively significant and it might be associated with its histological features. Among available staining and markers, Ki67 and AgNOR are considered as reliable ones for predicting clinical behavior of lesions. The aim of this study is to Evaluate the relationship between the expression of AgNOR and Ki67 with CGCG lesion's recurrence rate. The results can be useful for obtaining an accurate treatment plan for each individual case.

## 2 METHODS AND MATERIALS

### 2.1 Case selection

This study is a case‐control investigation based on 70 paraffin blocks of patients whoselesions are diagnosed with CGCG during the period of 2005–2020. These samples were collected from the archive of the pathology department of Shiraz School of Dentistry. By investigating patient's files and records, and updating their history by contacting them, 35 samples were selected with 15 recurrent cases and 20 nonrecurrent samples of CGCG. It was intended to collect the highest quantity of samples in both groups to provide higher reliability in results.

The aim of contacting the patients was to acquire verbal and written data and to make sure the information is updated if the lesion had recurred from the last visit. In one case the lesion had recurred more than once.

Excluding some of the samples based on different reasons, such as the thickness of the sample, necessary patient's details on their files and availability of the patient (to be contacted) was performed.

The ethical committee of Alborz Medical Science University confirmed this research with Code number IR.ABZUMS.REC.1401.079. All of the samples were stained by hematoxylin and eosin (H&E) staining method and then analyzed by two oral pathologists. Operators were blind to the sample's status to ensure the collecting data is accurate and reliable.

### 2.2 Ki67 marker staining and interpretation

The formalin‐fixed and paraffin‐embedded blocks preparation was performed by slicing them into sections with 4‐μ thickness, and performing tissue deparaffinization and rehydration. To implement IHC staining, Envision labeled peroxidase system (DAKO) was used. Then, Antigen retrieval was carried out by DAKO cytomation target retrieval solution for 20 min. After that, for blocking endogenous peroxidase activity, 3% H_2_O_2_ was applied. At first, the tissue sections were incubated with anti‐Ki67 primary antibody (ready to use, DAKO Corporation). Then, the sections were rinsed with phosphate buffered saline (PBS), and incubated with Envision solution. The chromogen that was used in this procedure is 3,3 di‐amino benzidinic (DAB). Finally, The sections were counterstained with Myer's Hematoxillyne (Shahela et al., 2013).

**Figure 1:**
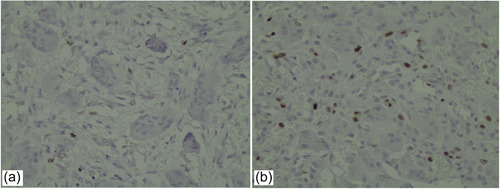
Ki67 marker (×400) (a) nonrecurrent lesion, (b) recurrent lesion.

Counting stained cells was carried out with Microscope Olympus, BX43F, Tokyo, at ×400 magnification (hpf). Cells were counted in 10 microscopic fields (Figure 1). The assessment of mononuclear stained proliferation was performed by counting the quantity of cells stained with Ki67 marker. The stained cells were considered nuclear staining. Ki67 marker only stains mononuclear stroma cells; so giant cells do not stain. To confirm the reliability of the staining technique, Burkitt Lymphoma cells were used as a positive control during the experiment. Eventually, the rate of the marker was defined by the mean of 10 microscopic field numerals. The results were represented in Table format (Razavi & Yahyaabadi, 2018). Parallel sections from which either the primary or secondary antibodies were excluded were considered as negative control.

In addition, the intensity of Ki67 proliferative marker was assessed separately. For mononucleolar stained cell count, any brown nucleus without noticing its intensity was considered to be positive (Lindblom, 2000; Pan et al., 1998). Immunoreactivity was classified into four groups including: (0) ≤5% negative, (1) 6%–25% low proliferation, (2) 26%–50% moderate proliferation, and (3) 51%–100% high proliferation of the cells which were considered positive (Alves et al., 2004).

### 2.3 AgNOR marker staining and interpretation

For AgNOR staining, Ploton approach was done by preparing the blocks into 5‐μ sections (Ploton et al., 1986). Fixed slides were stained by the standard method including a final solution composed of one volume of 2% Gelatin in 1% formic acid and two volumes of 50% silver nitrate.

At first, the slides were oriented horizontally into petri dishes and 3–4 drops of prepared solution were added by a pipette. After covering the petri dishes were with glass, they were heated up to 45°C for 30 min and retained in the darkness.

Thus, the glass cover was removed and the slides were rinsed by normal saline. They were dehydrated to xylene and fixed in a synthetic medium. Brown or black dots are considered NOR‐stained regions. To confirm the accuracy of the staining technique and its specificity, Breast Carcinoma cells were used as positive controls.

Negative controls included parallel sections from which either the primary or secondary antibodies were excluded.

Assessment of stained nucleolar organizer regions was based on the criteria that was proposed by Crocker et al.: for counting the stained nucleolar organizer regions in uni or multinucleated cells, a ×100 oil immersion lens was used in all samples (Crocker et al., 1989).

**Figure 2:**
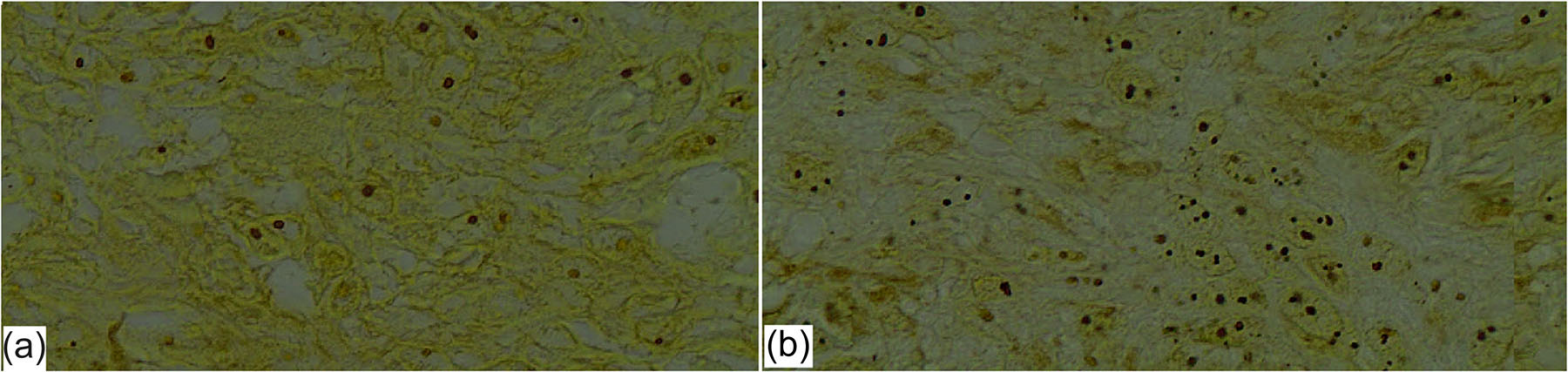
AgNOR staining (×1000) (a) nonrecurrent lesion, (b) recurrent lesion.

For every slide, 10 fields were randomly selected. In each cell, the brown or black stained regions inside the nucleus were recorded (Figure 2). Connected stains and stains located on the nucleolus were counted as one stained region. Regions with necrotized areas, severe inflammation, and artifacts were excluded (Sadri et al., 2010).

### 2.4 Data analysis

Selected samples, including 35 paraffin blocks of patients diagnosed with CGCG, were investigated to evaluate the relationship between the expression of AgNOR and Ki67 with the recurrence rate in CGCGs. The data were collected and analyzed using SPSS software, version 24.

The data were analyzed by statistical tests including chi‐square, Fisher, and *T*‐test; with the significance level of .05.

## 3 RESULTS

Among the studied cases, 40% of patients (14 patients) had recurrent lesions. In one patient the lesion had recured twice.

### 3.1 Demographic results

According to data analysis, 51.4% of all patients (18 patients) were male and 48.6% of them (17 patients) were female.

Based on the chi‐square test and *p*‐value (*p* = .89 > .05), there is not a significant difference between gender and recurrence rate. In patients with nonrecurrent lesions 52.4% (11 patients) were male and 47.6% (10 patients) were female and in patients with recurrent lesions, 50% (seven patients) were male and 50% (seven patients) were female.

According to the *T* and *p*‐values (<.05), there is a significant difference between age and recurrence rates. Based on mean data, recurrence is more often in younger patients (Table 1).

**Table 1:** Relationship between central giant cell granuloma (CGCG)'s recurrence rate and age.

*p*	Standard deviation	Mean	The eldest	The youngest	Recurrence
.046	13.9	31.28	55	6	Nonrecurrent
	9.1	22.5	44	12	Recurrent
	12.8	27.7	55	6	Total

### 3.2 Results for association between lesion's recurrence and its location

The site of the lesion in 20% of cases was maxillary bone, and in 80% of them, was mandible. According to the Fisher test and its *p*‐value (*p* = .67 > .05), there is not a significant difference between lesion's site and recurrence rate (Table 2).

**Table 2:** Association between side of central giant cell granuloma (CGCG)'s location and its recurrence rate.

Recurrent			Nonrecurrent		Recurrency
*p*	Percentage	Abundance	Percentage	Abundance	Location
.73	42.8	6	47.4	9	Right
	28.6	4	36.8	7	Left
	28.6	4	15.8	3	Bilateral
.67	14.3	2	23.8	5	Maxilla
	85.7	12	76.2	16	Mandible

The site of location in 45.5% of cases was in right sides of their jaws and in 33.3% of cases was in left side. In 21.2% of patients both sides were included. Based on Fisher test and its *p*‐value (>.05), there is not a significant difference between side of lesion's location and its recurrence rate (Table 2).

From another aspect in 30.3% of studied cases, the lesion was located in anterior area and in 45.5% of cases it was located in posterior area. In 24.2% of cases, both areas were included.

According to Fisher test and its *p*‐value (*p* = .6 > .05), there is not a significant difference between lesion's area and its recurrence rate.

By comparing treatment alternatives, it is concluded that 57.1% of patients that were treated by surgery (six patients out of 20) and 42.9% of patients that had hybrid treatment (seven patients out of 15), experienced recurrency. Hybrid treatment is considered to apply both surgery and corticosteroid therapy. Based on chi‐square test and *p*‐value (*p* = .16 > .05), there is not a significant difference between treatment approach and recurrence rate.

Of these 35 patients, 2.9% had Hyperparathyroidism in their history. According to Fisher test and its *p*‐value (*p* = .15 > .05), there is not a significant difference between Hyperparathyroidism history and lesion's recurrence rate.

### 3.3 Results for the association between ki67 marker and lesion's recurrence

Based on the Fisher test and its *p*‐value (<.05), there is a significant difference between Ki67 intensity and lesion's recurrence rate (Table 3) and it is concluded from the results that recured lesions had higher intensity grades. Among recurrent lesions, 64.3% lesions were considered as grade 2 and 28.6% lesions were considered as grade 1 for Ki67 intensity; while there were no grade 2 intensity among nonrecurrent lesions and 47.6% were considered as grade 1.

**Table 3:** Association between Ki67 intensity and lesion's recurrence rate.

Recurrent		Nonrecurrent		Recurrency
Percentage	Abundance	Percentage	Abundance	Intensity
7.1	1	52.4	11	0
28.6	4	47.6	10	1
64.3	9	0	0	2
*p* = .00				

According to the *T*‐test and its *p*‐value (<.05), there is a significant difference between the number of cells that are stained with Ki67 in two groups of patients with recurrent and nonrecurrent lesions. Results indicate that the number of stained cells is higher in patients with recurrency (Table 4).

**Table 4:** Assessment and comparison of the number of cells that are stained with markers based on recurrency in central giant cell granuloma (CGCG).

*p*	Standard deviation	Mean	The most	The least	Recurrency	Marker
.00	0.55	1.63	3.1	1	Recurrent	AgNOR
	1.05	4.12	5.3	2.3	Nonrecurrent	
.00	6.8	10.77	32.2	1.8	Recurrent	Ki67
	8.3	22.5	39.2	8.6	Nonrecurrent	

### 3.4 Results for association between AgNOR marker and lesion's recurrence

According to *T*‐test and its *p*‐value (<.05), there is a significant difference between the number of cells that are stained with AgNOR in two groups of patients with recurrent and nonrecurrent lesions. Results indicate that number of stained cells are higher in patients with recurrency (Table 4).

## 4 DISCUSSION

As it is mentioned before, CGCG is a benign lesion and issues like its prevalence or prognosis are not the main concern about this lesion. CGCG's tendency for recurrence is still a challenge (Richardson et al., 2022). Recurrence rate of CGCG can be different in aggressive and nonaggressive types and in lesions with more aggressive behavior, it is seen to be up to 70% (De Cidrac et al., 2021). Generally, lesions with more tendency of recurrence will be treated with more aggressive treatment procedures and complementary treatments can be considered, too.

It has been a while since clinical behavior and treatment prognosis of different lesions are determined according to their histological findings. Several studies have shown that CGCG's clinical behavior and its histological features are associated too (Melo‐Muniz et al., 2020).

In the present study, incidence rate of CGCG lesions was not different between two genders. Richardson et al. (2022) study's data is similar to the current study findings. But it is in controversy with the reported data from Shrestha et al. (2021) and Srivastava et al. (2022). In their studies, incidence rate was higher in females. Hormonal factors are considered as the reason for this finding. These controversies can be due to different sample size in each study.

Melo‐Muniz et al. (2020) propose that there is no relationship between recurrency rate of CGCG and gender. This finding was consistent with the results of our study.

Also in our data, CGCG's incidence age varies between 6 and 55 years old with an average of 27.7 which is consistent with Ramesh (2020) and Shrestha et al. (2021) reports. They have stated that the vast majority of CGCGs occur under 30. In addition, in a systematic review study, Srivastava et al. (2022) reported that 60% of their cases were younger than 30 years old.

According to the attained data from the present study, there was a meaningful relationship between patient's age and CGCG recurrency rate. It is concluded from the average measure that recurrence rate is higher in younger patients. Also Infante Cossío et al. (2007) and Melo‐Muniz et al. (2020) have mentioned in their studies that CGCG has a greater tendency to recur in younger patients; which is reasonable because there is a higher level of growth factors and higher growth potential in younger ages.

In addition, based on current study's data, prevalence of CGCG's incidence in mandible was higher than maxilla. Srivastava et al. (2022) stated that incidence of CGCG in mandible is twice as likely. Also, Shrestha et al. (2021) reported that incidence rate of CGCG in mandible is 70%.

From another aspect, lesions may occur in anterior or posterior portion. Determining the zone of anterior and posterior portion is based on the location of canine in each jaw. In our study, the area of the jaw between one canine to the other one is considered the anterior portion and the area that is located distal to that is considered the posterior portion. Contrary to the data reported from Ramesh (2020), occurrence of CGCG was more frequent in posterior portion of the present study.

According to our data, there was no statical difference between the location of the lesions and recurrency rate. It is consistent with proposed data from Melo‐Muniz et al. (2020) study which is relevant regarding the same recurrence‐related factors in each lesion's location.

Also, in the data attained from our case studies, there was no meaningful relationship between treatment plan and recurrency rate. In Shrestha et al. (2021) study, all of the patients were treated by resection and none of them reported a recurrency. On the other side Chrcanovic et al. (2018) by investigating 2270 cases in a systematic review, reported that among different approaches including enucleation, curettage, and resection, the lesions treated by resection had the minimum recurrency rate and the lesions treated by enucleation had the most tendency to recur. The controversy between the current study and Chrcavonic et al. can be a consequence of smaller sample size.

One of the most common indicators for assessment of cell proliferation is Ki67, which is suitable for determining cell's growth fraction (HM El Attar & Wahba, 2016). Since Ki67 can be detected during all active phases of the cell cycle and it is absent during resting phase, it can be helpful for determining cell proliferation (Scholzen & Gerdes, 2000). The current study has evaluated the relationship between two parameters of Ki67 and recurrence rate of CGCG; including Ki67 intensity and the number of cells that are stained with Ki67 marker. Our data indicated that there was a significant difference between both of these two parameters and recurrence rate of CGCG. Ki67 intensity and count was significantly higher in recurrent lesions group rather than nonrecurrent lesions.

In contrary, Kujan et al. (2015) used immunohistochemistry evaluation to determine the nature of multinucleated and mononuclear cells from PGCG, CGCG, and GCT (giant cell tumor) of long bones and to determine whether there is a correlation between recurrence and some markers including Ki67. They stated that the percentage of Ki‐67 positive mononuclear cells in PGCG was significantly higher than that of both CGCG and GCT but they found that there is no statistical correlation between recurrence and Ki67 marker.

In another study, Lujic et al. (2016) evaluated the role of Ki67 and some other markers in GCT's recurrency. They proposed that Ki67 is competent to be considered as a predicting index for GCT lesions recurrence.

Since in most articles, CGCG lesions are categorized as aggressive and nonaggressive, and aggressive lesions are considered as lesions with recurrency, following conclusions compare recurrent and nonrecurrent cases.

HM El Attar and Wahba (2016) compared Ki67 expression in two groups of aggressive and nonaggressive CGCG lesions. In their study, Ki67 was detected in all of aggressive and nonaggressive samples but it is significantly higher in aggressive variants.

Also in a comparative study, Razavi and Yahyaabadi (2018) evaluated clinicopathological features and immunohistochemical aspect of aggressive and nonaggressive CGCG lesions using Ki67 and CD31 marker. They found a relationship between clinical and immunohistochemical features and reported that difference between Ki67 expression in aggressive and nonaggressive lesions was noticeable.

In contrary, Melo‐Muniz et al. (2020) did not mentioned any statical difference between aggressive and nonaggressive CGCG lesions when they assessed immunohistochemical parameters including Ki67 to distinguish between aggressive and nonaggressive ones.

Since there was no study that adequately evaluate the relationship between Ki67 expression and CGCG's recurrency, we investigated articles of other oral lesions to illustrate prognostic value of Ki67 at predicting oral lesions recurrency.

Acikalin et al. (2004) evaluated prognostic value of Ki67 immunostaining in laryngeal SCC (squamous cell carcinoma) and stated that there is a strong relationship between Ki67 count and the lesion's recurrency.

Also, Sundberg et al. (2021) reported that expression of Ki67 was higher in recurrent oral leukoplakia rather than nonrecurrent ones.

Several studies have shown that AgNOR staining is capable to be used for diagnostic and prognostic purposes, considering criteria like mean AgNOR count or their distribution in the nuclei (Ray et al., 2003). The results of the present study showed that there was a meaningful relationship between AgNOR staining count and recurrency of CGCG lesions. AgNOR count was significantly higher in recurrent lesions group rather than nonrecurrent lesions.

Kashyap et al. (2014) performed computer assisted histomorphologic comparison on expression of AgNORs in the oral CGCG and PGCG and GCT of long bone. Analyzed data showed significant differences among various histological parameters between CGCG, PGCG, and GCT but there was no statistical correlation between expression of AgNORs in multinucleated giant cells and mononuclear cells among CGCG, PGCG, and GCT.

By investigating different databases, no article was found that evaluates the relationship of AgNOR expression and recurrency of CGCG lesions. Considering the fact that AgNOR staining is able to predict recurrency of all oral lesions such as SCC and OKC (odontogenic keratocyst), it was decided to assess this relationship.

Chandrashekar et al. (2020) analyzed recurrence of OKC by AgNOR and two other markers. OKC is a developmental odontogenic cyst which is considered as a benign cystic neoplasm but it can behave aggressively and it has a propensity to recur (Chandrashekar et al., 2020). This study stated that there is a significant difference between recurrent and nonrecurrent group and AgNOR expression is higher in recurrent lesions. Also, they suggested that further surgical interventions can be beneficial in patients with higher AgNOR expressions to acquire more promising prognosis. In another study, Teixeira et al. (1996) evaluated AgNOR staining in predicting recurrence‐free interval and stated that stained areas are capable of predicting increased risk of lesion's recurrency. Also Pillai et al. (2005) evaluated significance stained areas with AgNOR in early diagnosis and prognosis of oral SCC. Considering the fact that the majority of case studies experienced recurrency and in most of them T staging did not indicate prognosis correctly, they performed more histopathological assessments they figured out that AgNOR staining is significantly related to treatment outcomes and prognosis.

As it is mentioned before, both Ki67 and AgNOR are capable of indicating cell proliferation. By evaluating expression of these markers in different giant cell lesions, Studies have shown that Ki67 and AgNOR are also beneficial in assessing cell proliferation of these lesions.

Souza et al. (2000) compared the expression of these markers in PGCG and CGCG. Results indicated that mean count of Ki67 is higher in PGCG rather than CGCG but there was no significant difference in AgNOR count.

In addition, Selvi et al. (2012) evaluated the difference of Ki67 and AgNOR expression in recurrent and nonrecurrent OKC. Expression of these markers were significantly higher in recurrent lesions. In another study, Gawande and Chaudhary (2021) assessed the utility of actual proliferation index measured by expression of Agnor and Ki67 as a modality for prediction of recurrence in histopathologically negative surgical margins of oral SCC. The results indicated that the mentioned index is capable of predicting recurrence in oral SCC lesions. Also Titinchi (2022), in a systematic review article, formulated a recurrence risk stratification by Ki67 and AgNOR and other parameters such as age and size of the lesion. By investigating several studies, they stated that there is a strong relationship between expression of Ki67 and AgNOR and tendency of OKC for recurrence.

In conclusion, based on extracted data from different studies, it was determined that Ki67 and AgNOR markers are capable of predicting recurrency in SCC, leukoplakia, OKC, and other oral lesions. In the current study, their relationship with recurrency of CGCG lesions is evaluated which indicates that there is a meaningful relationship between them.

To mention the limitations of this study, there was limited number of samples available. In future studies and larger samples, dividing patients in specific groups including age and other markers can be beneficial.

## 5 CONCLUSION

Taking into consideration the data attained from studying histological features of AgNOR staining and Ki67 marker, these markers are beneficial for predicting recurrence tendency of CGCG lesions. There was a relationship between both Ki67 intensity and count, and recurrency rate of CGCG lesions. There was also a relationship between AgNOR staining count and incidence of recurrence. No correlation was found between both treatment plan and gender, and recurrence rate. Although, a relationship between younger age and incidence of recurrence was conducted. Further studies can be helpful in providing more evidence for the value of these markers in predicting CGCG lesions recurrency and also in evaluating efficiency of other biomarkers.

## AUTHOR CONTRIBUTIONS

Azadeh Andisheh‐Tadbir contributed to sample collection and investigating histopathological features. Arezoo Aghakouchakzadeh and Mina T. B. Dareh contributed equally to all of the procedures.

## CONFLICT OF INTEREST STATEMENT

The authors declare no conflict of interest.

## Data Availability

The data that support the findings of this study are available from the corresponding author upon reasonable request.
